# Morphology, Nucleation, and Isothermal Crystallization Kinetics of Poly(Butylene Succinate) Mixed with a Polycarbonate/MWCNT Masterbatch

**DOI:** 10.3390/polym10040424

**Published:** 2018-04-10

**Authors:** Thandi P. Gumede, Adriaan S. Luyt, Ricardo A. Pérez-Camargo, Agnieszka Tercjak, Alejandro J. Müller

**Affiliations:** 1 Department of Chemistry, University of the Free State (Qwaqwa Campus), Private Bag X13, Phuthaditjhaba 9866, South Africa; tpgumede66@gmail.com; 2 Center for Advanced Materials, Qatar University, P.O. Box 2713, Doha, Qatar; 3 POLYMAT and Polymer Science and Technology Department, Faculty of Chemistry, University of the Basque Country UPV/EHU, Paseo Manuel de Lardizabal 3, 20018 Donostia-San Sebastián, Spain; ricardi_perez001@ehu.es; 4 Group “Materials + Technologies” (GMT), Department of Chemical and Environmental Engineering, Faculty of Engineering, Gipuzkoa, University of the Basque Country UPV/EHU, 20018 Donostia-San Sebastián, Spain; agnieszka.tercjaks@ehu.es; 5 IKERBASQUE, Basque Foundation for Science, 48013 Bilbao, Spain

**Keywords:** PBS, PC/MWCNTs masterbatch, nanocomposites, morphology, nucleation, conductivity, isothermal crystallization

## Abstract

In this study, nanocomposites were prepared by melt blending poly(butylene succinate) (PBS) with a polycarbonate (PC)/multi-wall carbon nanotubes (MWCNTs) masterbatch, in a twin-screw extruder. The nanocomposites contained 0.5, 1.0, 2.0, and 4.0 wt% MWCNTs. Differential scanning calorimetry (DSC), small angle X-ray scattering (SAXS) and wide angle X-ray scattering (WAXS) results indicate that the blends are partially miscible, hence they form two phases (i.e., PC-rich and PBS-rich phases). The PC-rich phase contained a small amount of PBS chains that acted as a plasticizer and enabled crystallization of the PC component. In the PBS-rich phase, the amount of the PC chains present gave rise to increases in the glass transition temperature of the PBS phase. The presence of two phases was supported by scanning electron microscopy (SEM) and atomic force microscopy (AFM) analysis, where most MWCNTs aggregated in the PC-rich phase (especially at the high MWCNTs content of 4 wt%) and a small amount of MWCNTs were able to diffuse to the PBS-rich phase. Standard DSC scans showed that the MWCNTs nucleation effects saturated at 0.5 wt% MWCNT content on the PBS-rich phase, above this content a negative nucleation effect was observed. Isothermal crystallization results indicated that with 0.5 wt% MWCNTs the crystallization rate was accelerated, but further increases in MWCNTs loading (and also in PC content) resulted in progressive decreases in crystallization rate. The results are explained by increased MWCNTs aggregation and reduced diffusion rates of PBS chains, as the masterbatch content in the blends increased.

## 1. Introduction

Recently, environmental pollution and resource crises related to fossil-based polymers have led to the development of biodegradable polymers with similar functionality as petrochemical polymers, but that are readily susceptible to microbial action [1,2,3]. Aliphatic polyesters are representatives of biodegradable polymers and they are considered to be high performance, environmentally friendly biodegradable plastics [4,5]. One of these aliphatic biodegradable polymers is poly(butylene succinate) (PBS), known under the trade name “Bionolle^®^”. PBS is synthesized through the polycondensation reaction of glycols, such as ethylene glycol and 1,4-butanediol, and aliphatic dicarboxylic acids, such as succinic acid and adipic acid. This biodegradable polymer exhibits physical and mechanical properties closely comparable to those of the widely-used polyethylene (PE) and polypropylene (PP) [1,4,6]. PBS has many interesting properties such as biodegradability, melt processability, and thermal and chemical resistance. It can be processed in the field of textiles through melt blowing, multifilament, monofilament, nonwoven, flat, and split yarn, and also in the field of plastics through injection-molded products. However, other properties of PBS, such as softness, low melt viscosity and strength, as well as weak gas barrier properties, are often not sufficient for its further processing and end-use applications [3,5,7,8,9,10].

Incorporation of conductive carbon-based nanofillers, such as carbon nanotubes (CNTs), is an effective approach for improving the properties of PBS, provided that the CNTs are well dispersed in the polymer matrix. It is well known that the as-received CNTs tend to form agglomerates during mixing with polymers due to the strong van der Waals attraction between the carbon nanotubes [11]. In polymer/CNTs nanocomposites, aggregation of CNTs may become a defect and cause the mechanical properties of the composite to deteriorate. This problem can be overcome by using functionalized carbon nanotubes [12,13], which can provide multiple bonding sites to the polymer matrix so that the load can be transferred to the CNTs and inhibit the separation between the surfaces of the polymer and the CNTs. Another approach is to use a masterbatch, which involves the direct encapsulation of the CNTs into a polymer matrix, and the release of the CNTs to the other component in the nanocomposites that takes place during heating [3,4,14,15,16].

Several chemical methods have been reported for the functionalization of CNTs, such as (i) the use of *N*,*N*’-dicyclohexylcarbodiimide (DCC) to introduce a long alkyl chain onto multi-walled CNTs (MWCNTs), and (ii) surface wrapping of poly(sodium 4-styrenesulfonate) (PSS) with the aid of ultrasound [3,4]. In these studies, the MWCNTs showed a very low percolation threshold between 0.1 and 0.3 wt% MWCNTs, indicating the formation of conductive pathways in the PBS matrix. This indicates that the MWCNTs were well dispersed in the PBS matrix and showed better interfacial adhesion with the PBS phase than with each other, due to π–π interactions between the benzene rings, and between the long alkyl chain groups and the graphite rings of the MWCNTs.

A commercially available polycarbonate (PC)/MWCNTs masterbatch has been used as a filler and added it to the PC matrix in the subsequent melting process [14,15]. In this case, the MWCNTs were well separated and uniformly distributed in the PC matrix, and the mechanical properties showed significant improvements in the storage modulus in comparison to that of the neat PC matrix. In our previous work [17], PCL was melt blended with a PC/MWCNTs masterbatch, and the results showed partial miscibility, where two phases were formed (PC-rich and PCL-rich phases). Only a small number of MWCNTs diffused from the PC-rich phase to the PCL-rich phase. There was a fair increase in the nucleation efficiency and overall crystallization rate of PCL, as well as the thermal conductivity of the nanocomposite, with increasing MWCNTs content, but the tensile properties reduced or showed little change. Amongst the different methods used for dispersing MWCNTs, the masterbatch approach is the most favoured method, because it involves a solvent-less procedure that is beneficial in preserving the environment. The masterbatch method using PC as the main component could be useful in the dispersion of multi-walled carbon nanotubes in a PBS matrix, although there is currently no published information on PBS/PC blends, as far as the authors are aware.

In spite of the lack of information related to PBS/PC blends, it is known that PBS has a biodegradable character and the use of CO_2_ as one of the monomers in the preparation of polycarbonates would not only partially get rid of the dependence on petroleum but also provide a new approach in reducing the massive emission of CO_2_, which contributes to the greenhouse effect [18]. Therefore, the PBS/PC blends represent an interesting approach, and it is expected that such blends in combination with MWCNTs might lead to the development of interesting materials.

In this paper, MWCNTs were dispersed into a PBS matrix by melt-mixing PBS with a PC/MWCNTs masterbatch. The structure and properties of the nanocomposites were correlated with the dispersion, morphology, and nucleating effect of the MWCNTs on the PBS matrix. Additionally, the efficiency of the nucleation and the crystallization kinetics of the PBS component were determined by self-nucleation and isothermal crystallization studies.

## 2. Experimental

### 2.1. Materials

A commercial poly(1,4-butylene succinate) (PBS), extended with 1,6-diisocyanatohexane, was purchased from Sigma-Aldrich (Johannesburg, South Africa). It has a density of 1.3 g·cm^−3^ at 25 °C and a melting temperature of 120 °C. The weight-average molecular weight (*M_w_*) of PBS was 63,000 g·mol^−1^ [19].

A conductive masterbatch based on 85 wt% low-viscosity polycarbonate (Makrolon^®^ 2205 grade, *M_w_* of 20,100 g·mol^−1^ [20]) loaded with 15 wt% of MWCNTs (industrial grade NC7000), was obtained from Nanocyl (Sambreville, Belgium). It has a density of 1.175 g·cm^−3^. The average diameter and length of the MWCNTs were respectively 10 nm and 3–4 µm. The MWCNTs contained more than 90 wt% carbon and less than 10 wt% metal oxide impurities.

The nanocomposites were prepared by melt-mixing in a twin-screw extruder (Thermo Scientific HAAKE Mini Lab II (Thermo Fischer Scientific, Waltham, MA, USA) at the University of Pretoria, South Africa) operated under compressed air (100 rpm, 160 °C, 10 min). After extrusion, the samples were compression-moulded at 160 °C for 5 min under 50 kPa using a hydraulic melt press. The calculated weight percentages of the different components in each of the investigated nanocomposites are given in Table 1.

### 2.2. Sample Characterization

Scanning electron microscopy (SEM) analyses were performed using a TESCAN VEGA 3 (Brno, Czech Republic) scanning electron microscope. The samples were sputter-coated with gold for 60 s to produce conductive coatings onto the samples. The acceleration voltage used was 15 kV.

Atomic force microscopy (AFM) experiments were performed for the 93/(6/1) and 73/(23/4) *w*/*w* PBS/(PC/MWCNTs) samples at room temperature using a Bruker Multimode 8 scanning probe microscope equipped with a Nanoscope V controller. The micrographs, the sizes of which were in the range of 1.0–14.1 μm, were obtained in tapping mode by using microfabricated silicon tips/cantilevers (cantilever spring constant, *k* = 42 N·m^−1^, and resonance frequency, *f*_0_ = 320 kHz, Bruker, Santa Barbara, CA, USA). Height and phase AFM images of lamellae and MWCNTs were collected simultaneously and subjected to a first-order plane-fitting procedure to compensate for the tilt. The height and phase AFM images were similar, and consequently in this work only the phase AFM images will be reported. To obtain cross-sectional AFM images, the samples were cut using an ultramicrotome Leica Ultracut R with a diamond blade.

Simultaneous small-angle X-ray scattering (SAXS) and wide-angle X-ray scattering (WAXS) experiments were performed at the beamline BL11-NCD, ALBA Synchrotron facility in Barcelona, Spain. Each sample was placed in a DSC pan, and was put on a Linkam THMS600 (Linkam Scientific Instruments, Tadworth, Surrey, UK) hot stage coupled to a liquid nitrogen cooling system. The hot stage was programmed to perform cooling and subsequent heating and at the same time measurements of the SAXS/WAXS patterns were taken. The thermal protocol was as follows: heating from room temperature to 180 °C, followed by a holding step of 3 min. Once the thermal history was erased, the samples were cooled down at 50 °C min^−1^ to the selected isothermal temperature. Different isothermal times were used depending on the temperature. Finally, after the isothermal step, the samples were heated at a rate of 5 °C min^−1^. The energy of the X-ray source was 12.4 keV (*λ* = 1.0 Å). In the SAXS configuration, the sample-detector used was an ADSC Q315r detector with a resolution of 3070 × 3070 pixels, a pixel size of 102 μm^2^, and a distance of 6495.0 mm with a tilt angle of 0°, whereas in the WAXS configuration, the sample-detector was a Rayonix LX255-HS detector (Rayonix, Evanston, IL, USA) with a resolution of 1920 × 5760 pixels, a pixel size of 44 μm^2^, and a distance of 132.6 mm with a tilt angle of 21.2°. The intensity profile showed the plot of the scattering intensity as a function of the scattering vector, $q=4\pi \;sin\theta \;\lambda^{-1}$, where *λ* is the X-ray wavelength (*λ* = 1.0 Å) and *2θ* is the scattering vector. The scattering vector was calibrated using silver behenate (SAXS) and chromium (III) oxide (WAXS).

Differential scanning calorimetry (DSC) analyses were performed using a heat flux Perkin Elmer DSC 6000 (Akron, OH, USA) under nitrogen flow (flow rate 20 mL·min^−1^) to minimize degradation of the samples, and the instrument was calibrated at a heating rate of 20 °C·min^−1^ using the onset temperatures of melting of indium and zinc standards, and the melting enthalpy of indium. The sample weight was ~5 mg in all cases.

For the non-isothermal DSC analyses, the samples were melted in the DSC for 3 min at 270 °C to erase any previous thermal history. The samples were then cooled at 20 °C·min^−1^ from 270 to −20 °C, and then heated at the same rate from −20 to 270 °C.

The self-nucleation (SN) tests were performed according to a procedure established by Fillon et al. [21], and further developed and studied by Müller et al. [22,23,24]. The complete procedure is as follows:

(a) The sample was heated from 25 to 270 °C at 20 °C min^−1^ and maintained at that temperature for 3 min to erase thermal history.
(b) It was then cooled from 270 to −20 °C at 20 °C min^−1^ to create the initial “standard” state, and held at that temperature for 3 min.
(c) It was then heated from −20 °C to a selected self-nucleation temperature (*T_s_*), located in the final melting temperature range of the sample, and held at that temperature for 5 min.
(d) It was again cooled to −20 °C, where the effects of thermal treatment are reflected in the crystallization behaviour of the sample.
(e) Finally, it was heated to 270 °C, where the effects of thermal treatment are reflected in the melting behaviour of the sample.

The most important parameters during SN are: (1) the heating and cooling rates used, (2) the *T_s_* temperature, and (3) the time spent at *T_s_*.

The isothermal crystallization experiments were performed by following the procedure recommended by Lorenzo et al. [25], in which isothermal crystallization temperatures (*T_c_*) are chosen where no crystallization occurred during the cooling step from the melt (performed at 60 °C min^−1^). The samples were heated to 270 °C and kept at this temperature for 3 min to erase the thermal history. Then a controlled cooling at 60 °C min^−1^ was applied, down to the set isothermal *T_c_*. The sample was then kept at the set *T_c_* for a crystallization time (*t_c_*) until saturation was reached. Finally, the sample was heated from *T_c_* to 270 °C at 20 °C min^−1^, to record the melting behaviour of the isothermally crystallized sample.

To determine the equilibrium melting temperatures, $T_{m}^{o}$, of the samples, the final step in the isothermal crystallization procedure (i.e., heating at 20 °C min^−1^), was used to record the melting of the crystals formed at different crystallization temperatures, *T_c_*. Hoffman–Weeks extrapolation [26] was then applied by plotting the observed melting temperature (*T_m_*_(*obs*)_) against *T_c_* to observe the intersection of this line with another line with a slope equal to 1 (*T_m_* = *T_c_*), which represents the thermodynamic equilibrium.

Dynamic mechanical analyses (DMA) were performed in a Perkin Elmer Diamond DMA (Akron, OH, USA) from −100 °C to the onset of melting of PBS, which is ~100 °C, in the bending (dual cantilever) mode at a heating rate of 3 °C min^−1^ and a frequency of 1 Hz.

The tensile analysis of the samples was carried out using an Instron 4301 universal testing machine (Instron, Norwood, MA, USA) at a cross-head speed of 10 mm min^−1^. The dumbbell shaped samples had a Gauge length of 20 mm, a thickness of 1 mm and a width of 5 mm. The samples were tested at a controlled ambient temperature of 23 °C and 50% relative humidity. Three samples of each composition were tested and average values with standard deviations are presented.

Thermal conductivity measurements were performed using a Therm Test Inc. Hot Disk TPS 500 thermal constant analyser (Fredericton, NB, Canada). The instrument uses the transient plane source method. A 3.2 mm radius Kapton disk type sensor was selected for the analysis. The sample discs were 5 mm thick and 12 mm in diameter. The sensor was placed between two sample discs of the same composition. The measurements were made for a period of 25 s in order to prevent the heat flow from reaching the boundary of the samples. Five measurements were performed for each composition. The thermal conductivities are reported as average values with standard deviations.

## 3. Results and Discussion

### 3.1. Miscibility Assessment

The glass transition temperature (*T_g_*) is used to determine the interaction between the components of a polymer blend. The blend is considered completely miscible if only one *T_g_* is observed with its position determined by the composition of the blend. Two distinct *T_g_*s symbolize an immiscible blend with the *T_g_*s corresponding to those of the two-parent homopolymers. However, when the two polymers are partially miscible, there are still two *T_g_*s that will be shifted towards each other, with the degree of shift being dependent on both blend composition and degree of miscibility [27].

**Figure 1:**
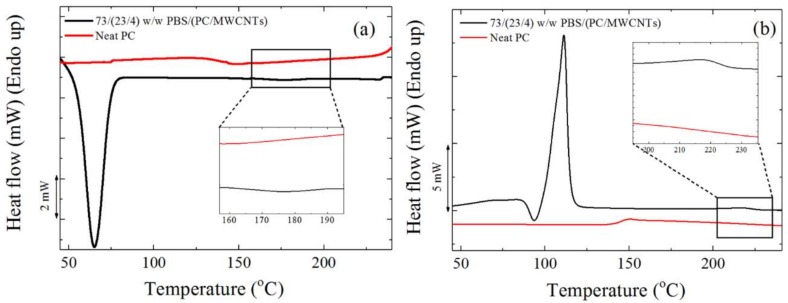
DSC (**a**) cooling and (**b**) second heating curves for neat polycarbonate (PC) and the 73/(23/4) *w*/*w* PBS/(PC/MWCNTs) nanocomposite. The zoomed regions correspond to the crystallization (see (**a**)) and melting (see (**b**)) of PC in the nanocomposites and its absence in the neat PC.

In the present study, the *T_g_* of the PC component in the nanocomposites could not be detected through either DSC or DMA because (i) PBS, the major component in the blends, melted at a temperature below the *T_g_* of PC (DMA analyses cannot be performed at temperatures above the *T_m_* of PBS), and (ii) PC, the minor component in the blends, crystallized around 180 °C, as can be seen from the melting and crystallization peaks in Figure 1. Since the PC crystallized, its *T_g_* was difficult to observe by DSC, as the amount of mobile amorphous fraction per unit mass was smaller in the blends.

**Figure 2:**
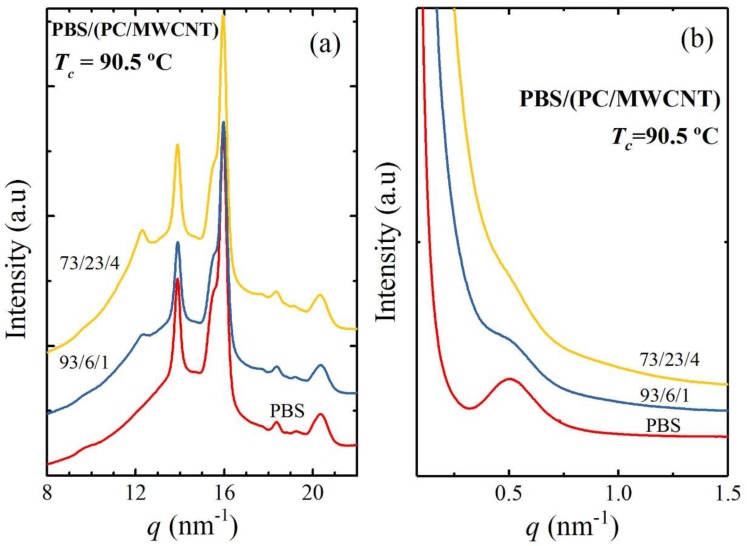
(**a**) WAXS diffractograms taken at a selected isothermal temperature of 90.5 °C; (**b**) SAXS patterns taken at the same temperature.

The crystallization of the PC component was also confirmed by simultaneous SAXS/WAXS analyses for the 93/(6/1) and 73/(23/4) *w*/*w* PBS/(PC/MWCNTs) nanocomposites. Figure 2 shows the final X-ray patterns taken at a selected isothermal crystallization temperature of 90.5 °C. The main WAXS reflections shown by neat PBS are visible in the X-ray patterns of the nanocomposites. The main reflection peaks of PBS are located at *q* values of 13.9 and 16.0 nm^−1^, and correspond to the (002) and (110) planes, respectively. The medium intense reflection at 15.5 nm^−1^, which corresponds to the (012) plane, as well as the minor reflections at 18.4 and 20.4 nm^−1^, which correspond to the (121) and (111) planes, also appear in all the samples. All the reflections are consistent with the reported monoclinic unit cell of *α*-PBS with unit cell parameters *a* = 5.232, *b* = 9.057 and *c* = 10.900 Å and *γ* = 123.87°.

**Figure 3:**
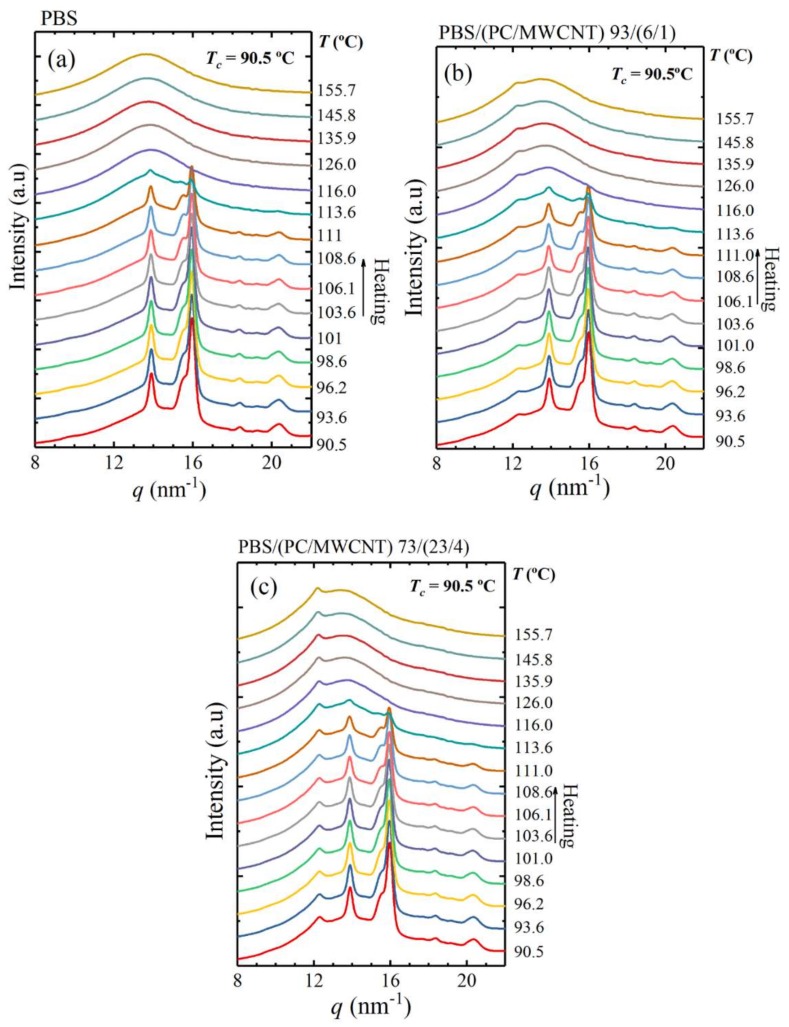
WAXS patterns taken during heating at 5 °C min^−1^ after the isothermal step at 90.5 °C for (**a**) neat PBS, (**b**) PBS/(PC/MWCNTs) (93/6/1), and (**c**) PB/(PC/MWCNTs) (73/23/4).

In addition to the PBS unit cell peaks, there is a peak at 12.3 nm^−1^ (equivalent to a 2*θ* of 17.4°), which appears in the nanocomposites and becomes more pronounced as the PC content in the nanocomposites increases. This peak corresponds to the PC component that is able to crystallize due to the plasticization effect of the PBS (see Figure 3). The same effect was observed in our previous work of PCL/(PC/MWCNT) nanocomposites at high PC contents [17]. Note that such a plasticization effect evidences the partial miscibility between the PBS and PC. Figure 2b shows the SAXS patterns taken at the same condition used in the WAXS experiments. In these patterns, the PBS signal observed for neat PBS decreased as the PC content increased, since the single peak corresponds mainly to the long spacing of PBS lamellae. However, at higher PC contents (i.e., 23 wt%), the PC is able to crystallize due to the plasticization effect of the PBS, and the SAXS signal is not clear due to the overlap of the long spacings generated by the lamellae of PBS and PC. The signal observed is probably an average of these two long spacings and is shifted to lower *q* values.

For the sake of clarity, the WAXS patterns were taken during heating after the isothermal step for some selected samples. Figure 3 shows that the PC peak does not disappear when the PBS is already molten at *T* > 116 °C. This behaviour is clearly observed at the higher PC content (Figure 3c). According to our results and the literature [28,29], PC is able to crystallize, as mentioned earlier in the discussion, due to the plasticization effect of PBS. As a result, it shows a main reflection at 2*θ* = 17.1° (12.34 nm^−1^). This capability of plasticization is an evidence of partial miscibility. This PC peak is in line with the one reported in our previous work of the PCL/(PC/MWCNTs) nanocomposite, in which the PCL also acted as a plasticizer for PC [17].

The *d*-spacings for all the reflections shown in Figure 2a were calculated from Equation (1). The long periods were calculated from the main PBS peaks in the SAXS patterns in Figure 2b. The relevant values are tabulated in Table 2.

(1) $$d^{*}=\frac{2\pi }{q_{max}}$$

The *d*-spacings and the d* values of neat PBS and the PBS in the nanocomposites are almost the same at low PC contents (i.e., 6 wt%). In the case of the 73/(23/4) *w*/*w* PBS/(PC/MWCNTs) nanocomposite, the peak related to the PC component is the same as the one reported in the literature (0.464 nm) [28]. In the SAXS patterns, an overlap between the long spacings of PC and PBS occurs and this explains the decrease in d* values compared to those for the other samples.

**Figure 4:**
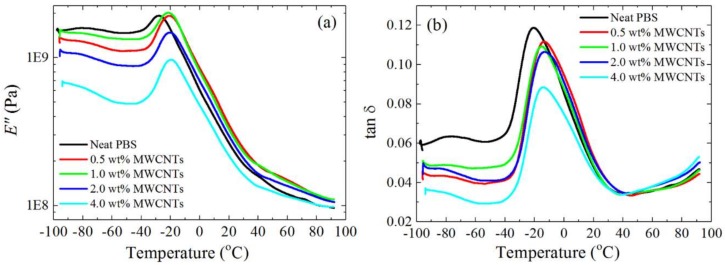
DMA (**a**) loss modulus (*E*″) and (**b**) tan *δ* curves for the investigated samples.

**Figure 5:**
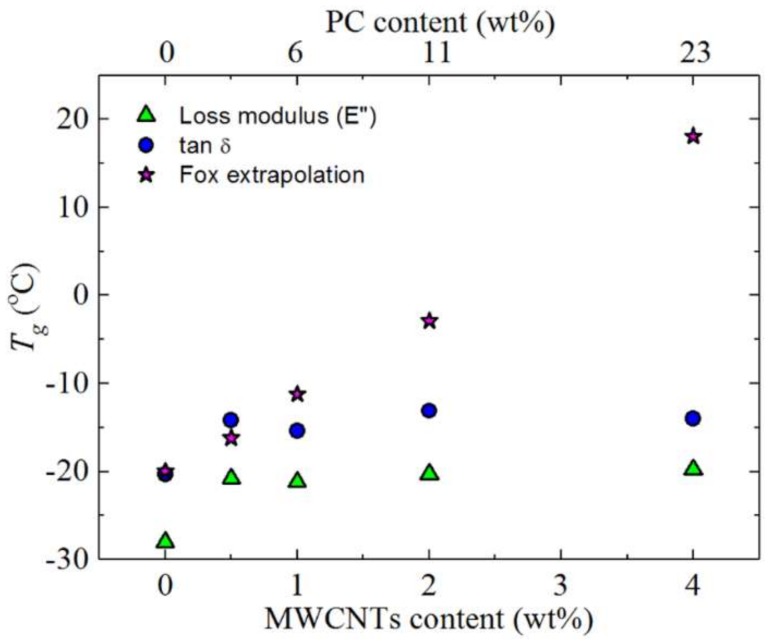
Glass transition temperatures of neat PBS and the PBS/(PC/MWCNTs) nanocomposites as a function of MWCNTs content (the corresponding PC content is indicated in the top x-axis). The Fox extrapolation *T_g_* data was obtained using the tan *δ* values.

Figure 4 shows the loss modulus (*E*″) and tan *δ* curves for neat PBS and PBS in the different nanocomposites. The *T_g_* values obtained from the loss modulus and tan *δ* curves are slightly different for the same sample, with *T_g_*_,*E”*_ < *T_g_*_,*tan δ*_ (Figure 5), as expected [15,30,31]. The trends from the different sets of results are, however, the same.

The results show that the *T_g_* of PBS in the nanocomposites is higher than that of neat PBS. It is well known that when two polymers are completely miscible, the *T_g_*s of the two polymers should change according to the trend predicted by the Fox equation (Equation (2)).

(2) $$\frac{1}{T_{g}}=\frac{w_{1}}{T_{g1}}+\frac{w_{2}}{T_{g2}}$$

where *T_g_* is the blend glass transition temperature, and *T_gi_* and *w_i_* are the respective glass transitions and weight fractions of the two blend components.

Figure 5 shows how the *T_g_* values change with composition. The *T_g_* increases (about 10 °C) upon addition of (6/1) *w*/*w* PC/CNTs, and then stays constant. The Fox equation only fits the change in *T_g_* for the sample with the lowest content of PC (i.e., 6 wt%). One possible explanation for the observed increase in *T_g_* is that the MWCNTs imposed restrictions on the molecular mobility of PBS [32]. However, since the *T_g_* does not change with increasing masterbatch content, this possibility can be ruled out.

On the other hand, partial miscibility between PC and PBS can explain the *T_g_* increase upon 6 wt% PC addition, as a PBS-rich phase develops. The lack of variation of *T_g_* with further increases in PC content may be related to a saturation effect of the amount of PC that can be dissolved in the PBS phase. Additionally, as in the PCL/(PC/MWCNTs) masterbatch case [17], the plasticization of PC by PBS led to PC crystallization. This, and the fact that the PBS melts at a temperature well below the *T_g_* of PC, made it difficult to observe the PC glass transition. Based on the presented data, we conclude that the blends are partially miscible and contain both PC-rich and PBS-rich phases in the limited composition range explored in this work (from 6 to 23 wt% PC content).

### 3.2. Morphology

SEM and AFM images for the 97/(2.5/0.5) and 73/(23/4) *w*/*w* PBS/(PC/MWCNTs) nanocomposites were obtained to confirm the presence of two phases (PBS-rich and PC-rich phases), and to observe whether MWCNTs diffused into the PBS phase.

**Figure 6:**
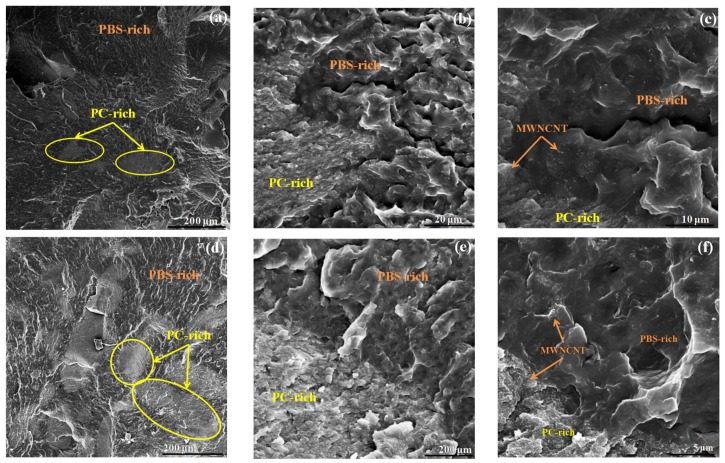
(**a**,**d**) Low and (**b**,**c**,**e**,**f**) high magnification SEM micrographs for the 97/(2.5/0.5) and 73/(23/4) *w*/*w* PBS/(PC/MWCNTs) nanocomposites. The yellow ellipses (see (**a**,**d**)) indicated the PC-rich phase. Figure 6b,e correspond to the interphase, whereas in c and f the PBS- and PC-rich phases as well as the position of MWCNTs in these phases are indicated.

It can be seen at low magnifications (Figure 6a,d) that there exists an interphase between the PBS-rich and the PC-rich phases, and that there are large ellipsoidal phases dispersed in the polymer matrix. The size of the dispersed phase (which corresponds to the PC-rich phase) is unexpectedly large considering the amount of the PC that has been added to the blends (i.e., 2.5 and 23 wt% for each blend examined in Figure 6), indicating a macro-phase segregation in the blends. In our previous work of PCL/(PC/MWCNTs) nanocomposites, the size of the dispersed phase was small, hence some of the MWCNTs were able to diffuse from the PC-rich phase to the PCL-rich phase [17]. In this case, the MWCNTs are mostly restricted within the ellipsoidal PC-rich phases. However, there are a certain number of nanotubes that can be observed in the PBS-rich phase (especially for the 0.5 wt% MWCNTs nanocomposite), when the micrographs are analysed in detail. Due to the limited miscibility between PC and PBS, only a small fraction of MWCNTs were able to migrate from the PC-rich phase to the PBS phase. The DSC results (to be presented below) also show nucleating effects of MWCNTs on the PBS-rich phase, which are present in the samples with low MWCNTs content. At higher contents, i.e., 4 wt% MWCNTs, the nucleation effect disappears, most probably as a result of MWCNTs agglomeration.

Further evidence for partial miscibility that can be observed in Figure 6, is the nature of the interphase between the PBS-rich and the PC-rich phases. There are no voids in between the phases (like in immiscible blends), but rather a smooth concentration gradient.

**Figure 7:**
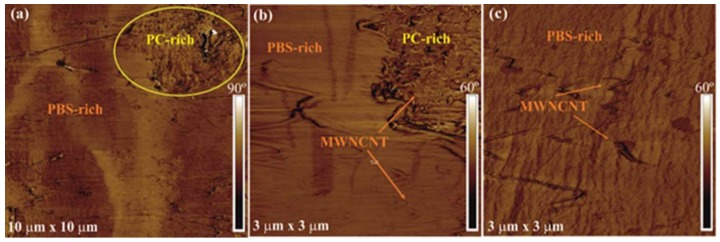
(**a**) Low and (**b**) high magnification AFM phase images for the 93/(6/1) *w*/*w* PBS/(PC/MWCNTs) nanocomposite, and high magnification AFM phase image of the PBS-rich matrix (**c**). The yellow ellipses (see (**a**)) indicate the PC-rich phase.

Figure 7 shows AFM phase images for the 1 wt% MWCNT nanocomposite at different magnifications. Similar to the SEM results, the low-magnification AFM image allows one to distinguish both PBS-rich and PC-rich phases (Figure 7a). To facilitate the recognition of both phases, the PC-rich phase is marked with a yellow ellipse. Moreover, as can be clearly seen at higher magnifications (Figure 7b), there exists a clear interphase between the PBS-rich matrix and the PC/MWCNTs masterbatch. The interphase reveals an intimate contact between the two phases, which is probably a consequence of the partial miscibility between the two polymers in the blends. Higher magnification (Figure 7b) images also indicate the presence of MWCNT not only in the PC/MWCNTs masterbatch phase, but also in the PBS-rich matrix. At higher magnifications, single MWCNTs are clearly visible in the PBS-rich matrix in the AFM phase image of the PBS-rich phase (Figure 7c).

### 3.3. Non-Isothermal DSC

**Figure 8:**
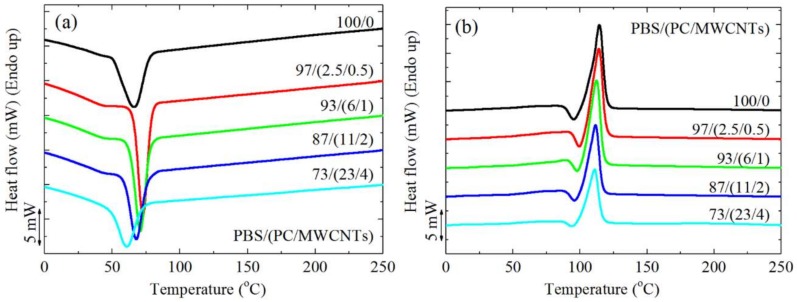
DSC (**a**) cooling and (**b**) second heating curves at 20 °C min^−1^ of neat PBS and the PBS/(PC/MWCNTs) nanocomposites.

Figure 8 shows the DSC (a) cooling scans after erasing the thermal history, and (b) the subsequent heating scans performed at 20 °C min^−1^ for the different investigated samples. All the investigated samples display a PBS-crystallization peak temperature (*T_c_*) around 60–75 °C (Figure 8a). In the subsequent heating scans shown in Figure 8b, neat PBS and the nanocomposites display a melting peak (*T_m_*) between 110 and 120 °C, and the melting peak is preceded by a cold crystallization peak at around 95 °C. This peak has been previously attributed to the recrystallization of partially melted thin lamellae of PBS [33].

**Figure 9:**
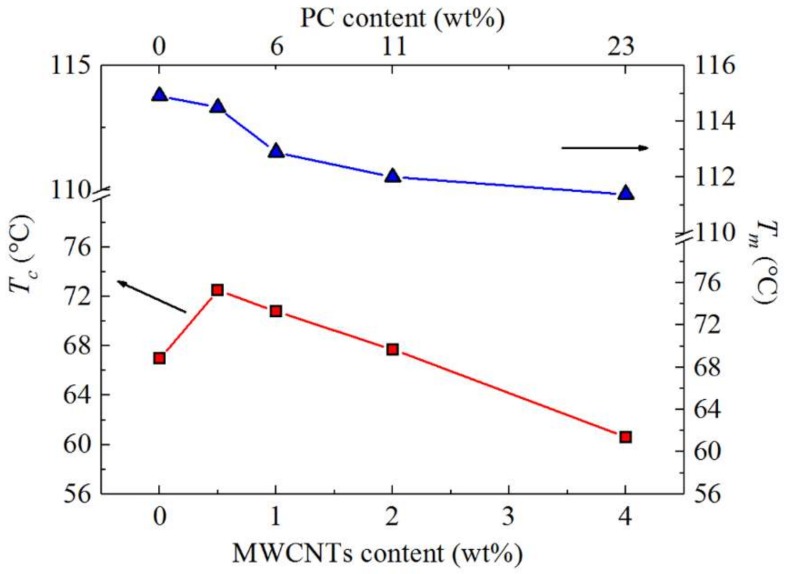
DSC crystallization and second heating melting temperatures as a function of MWCNTs content for neat PBS and the PBS/(PC/MWCNTs) nanocomposites (note that the PC content is indicated at the top x-axis).

To further examine the results presented in Figure 8, the *T_c_* and *T_m_* values were plotted as a function of MWCNTs as well as PC content in Figure 9. The *T_c_* of the PBS in the nanocomposites is higher than that of neat PBS up to 2 wt% MWCNTs, and the nucleation effect of the MWCNTs is a maximum for the 0.5 wt% MWCNTs composition. The MWCNTs diffusion to the PBS-rich phase should be most effective in this case because of the almost complete miscibility with the PC phase at this composition. At higher concentrations, there is a decrease in the nucleation effect as indicated by the decrease in *T_c_* values. This is probably due to the MWCNTs aggregation in the PC-rich phase, which restricted the MWCNTs from diffusing into the PBS-rich phase and participating in nucleating PBS. At a concentration of 4 wt% MWCNTs, an antinucleation effect appears, which can only be attributed to the migration of active nucleating heterogeneities from the PBS-rich phase to the PC-rich phase. At a composition of 4 wt% MWCNTs, the migration of MWCNTs to the PBS-rich phase did not occur, probably because of the higher amount of PC in the blend.

A melting point depression is observed for the PBS component with an increase in the masterbatch content (Figure 9), which would have been caused by the partial miscibility with the PC chains in the blend, in combination with the drop in *T_c_* values caused by the nucleation changes. The nucleating ability of the MWCNTs is quantified in the next section through self-nucleation experiments.

### 3.4. Self-Nucleation (SN)

Carbon nanotubes have been reported to act as nucleating agents for PBS [3,4,8,10,34,35], but their nucleating ability depends on the interaction between the polymer and the MWCNT surfaces. The maximum shift in *T_c_* reported in previous works for functionalized CNTs acting as nucleating agents was around 5 °C [3,8].

To evaluate the efficiency of the MWCNTs as nucleating agents, it is necessary to compare their effect with that of the PBS self-nuclei. Self-nucleation is a thermal protocol for the production of self-nuclei within a polymer melt, so that the nucleation density can be greatly increased. In theory, the best nucleating agent for any polymer is its own crystal fragments or chain segments with residual crystal memories [21,22,23,24]. The self-nucleation of PBS has already been studied and reported in the literature but for a different sample [36]. In the present work, a commercial PBS was used, and the self-nucleation experiments and their results are reported in the Supplementary Information (Figures S1 and S2).

**Figure 10:**
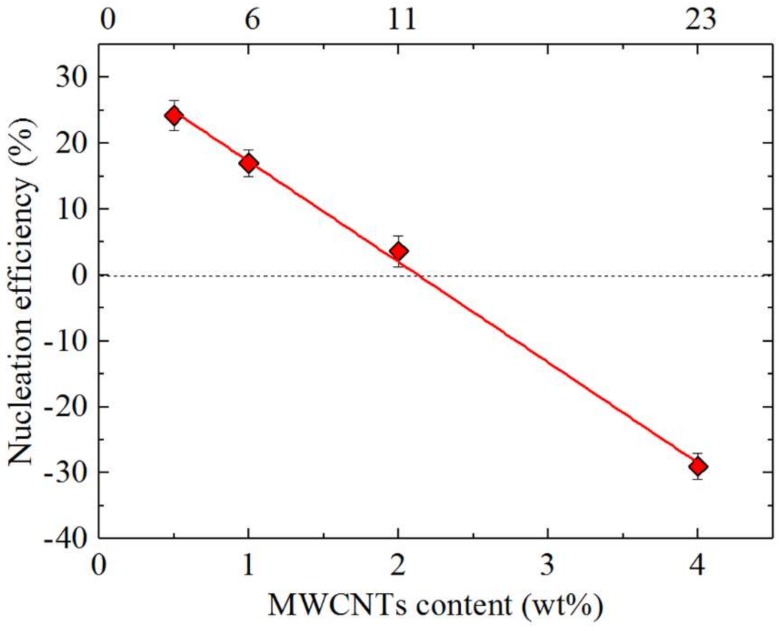
Nucleation efficiency as a function of MWCNTs content. The PC content is indicated in the top x-axis.

The efficiency of the MWCNTs as nucleating agents for the PBS matrix was calculated according to Equation (3), which was proposed by Fillon et al. [37].

(3) $$NE=\frac{T_{c,NA}-T_{c,PBS}}{T_{c,max}-T_{c,PBS}}\times 100$$

where *T_c_*_,*NA*_ is the peak *T_c_* value determined from the non-isothermal DSC cooling run for a sample of the polymer containing the nucleating agent (NA), *T_c_*_,*PBS*_ is the peak *T_c_* value for neat PBS after its crystalline history has been erased (67.0 °C) (also determined from the non-isothermal DSC cooling scan), and *T_c_*_,*max*_ is the maximum peak crystallization temperature determined after neat PBS has been self-nucleated at the ideal *T_s_* (89.3 °C, see the Supplementary Information for details on how to determine the ideal *T_s_* value.) [23,37]. Figure 10 shows the percentage nucleation efficiency of MWCNTs in the PBS/(PC/MWCNTs) nanocomposites.

It is observed in Figure 10 that the nucleation efficiency decreases with increasing MWCNTs content. This is consistent with Figure 9. At the highest MWCNTs content (i.e., 4 wt%), the nucleation efficiency is below 0%. This behaviour is unexpected, since negative nucleation efficiencies (i.e., antinucleation effect) have been reported to be primarily due to interactions between the polymer matrix and the nucleating agent, e.g., C-PCL/MWCNT-*g*-L-PCL [38], polylactide grafted cellulose nanocrystals (CNC-*g*-PLA) and poly(β-hydroxybutyrate) (PHB) [39], and PCL-*grafted*-lignin (with high lignin contents) system [40]. Generally, such interactions make the diffusion of the polymer matrix difficult. Some of these interactions are hydrogen bonding and threading effects, and it was also reported that the surface modified CNC particles retarded the heterogeneous nucleation of PHB crystals by restraining the relaxation of the neighbouring PHB chain segments.

In the work reported in this paper, there was no group in either the PBS matrix or the masterbatch that could cause similar interactions. Two possible reasons for this behaviour are therefore: (1) the MWCNT aggregation or (2) its encapsulation in the carrying polymer (i.e., the PC used in the masterbatch), which might lead to the saturation of the system, or to no or little contact between the MWCNTs and the PBS. In both cases, the expected behaviour is a nucleation efficiency equal to zero.

The significant decrease in the nucleation efficiency can only be explained by a complex behaviour in which a migration of heterogeneities from the PBS matrix to the PC in the masterbatch occurs, assisted by the previously reported plasticization effect (see Section 3.1). The MWCNTs are then confined to the PC, due to its crystallization, preventing the MWCNTs diffusion to the PBS matrix. Therefore, the PBS matrix has neither all the heterogeneities nor the MWCNTs, and as a result both its crystallization temperature as well as its crystallization kinetics decrease (see Section 3.5).

### 3.5. Overall Isothermal Crystallization Studied by DSC

**Figure 11:**
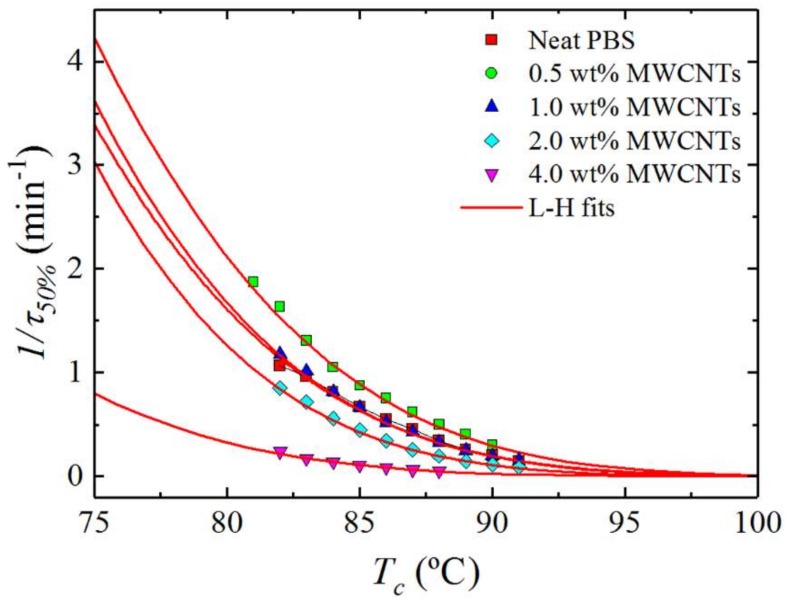
Overall crystallization rate (1/*τ*_50%_) as a function of isothermal crystallization temperature (*T_c_*) for neat PBS and for the PBS/(PC/MWCNTs) nanocomposites. The red solid lines represent fits to the LH theory.

The combined effect of MWCNTs and PC at different contents on the isothermal crystallization kinetics of PBS is presented in this section. Figure 11 shows the inverse of the half crystallization time (1/*τ*_50%_), which is an experimental measure of the overall crystallization rate, as a function of the isothermal crystallization temperature (*T_c_*) for neat PBS and the nanocomposites. The results are a complex function of the nucleation efficiency of MWCNTs (which depends on the composition as demonstrated in Figure 9 and Figure 10) and the partial miscibility with PC in the blends.

Figure 11 shows that the nucleation effect of the 0.5 wt% MWCNTs can impact on the overall crystallization kinetics (which includes both nucleation and growth), accelerating it despite the higher *T_g_* of the PBS-rich phase compared to that of neat PBS. For this composition, the nucleation effect can dominate the behaviour because of a possible depression in crystal growth which is a result of the reduced diffusion of the PBS chains within the PBS-rich phase (the diffusion of which would be affected by the miscibility with the more rigid PC chains). Increasing the content of MWCNTs to 1 wt% (and also the content of PC in the blend to 6 wt%) produces an equilibration effect between nucleation and growth that matches exactly the crystallization kinetics of neat PBS. Increasing the masterbatch content in the blends produces a decrease in the overall crystallization kinetics as the efficiency of nucleation decreases. The decrease in overall crystallization rate is particularly large for the blend with 4 wt% MWCNT, a reflection of the antinucleation effect previously discussed.

**Figure 12:**
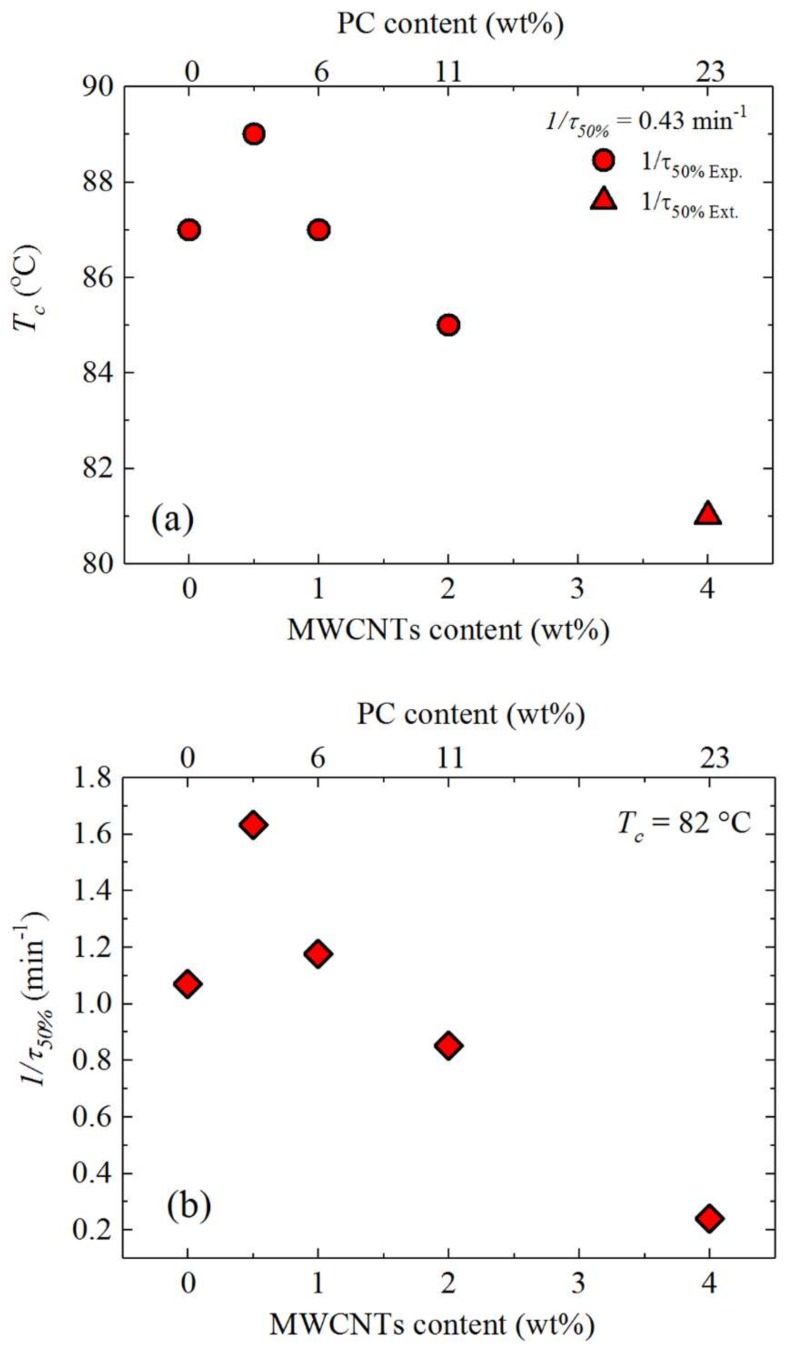
(**a**) Crystallization temperature as a function of MWCNTs content at constant 1/*τ*_50%_ = 0.43 min^−1^; (**b**) overall crystallization rate as a function of MWCNTs content at constant *T_c_* = 82 °C. The top x-axis indicates the PC content.

Another way to examine the results presented in Figure 11 is by taking the values of the crystallization temperature for which the blends reach a constant value of 1/*τ*_50%_ (i.e., 0.43 min^−1^) (Figure 12a) and the 1/*τ*_50%_ values at a constant *T_c_* (i.e., 82 °C) (Figure 12b), as a function of MWCNTs content. Figure 12a shows the experimental and extrapolated data using the Lauritzen and Hoffman (LH) theory, which is explained in detail in Section 3.5.2. The results are fully consistent with the non-isothermal results presented in Figure 9 and with the self-nucleation results of Figure 10. Figure 12a shows that in order to obtain a predetermined (arbitrarily chosen) constant crystallization rate, the nanocomposite with only 0.5 wt% MWCNTs needs a lower supercooling than neat PBS, but as the MWCNTs content increases (together with the PC content in the blends), the supercooling applied to obtain the same rate needs to be substantially increased, even beyond that needed by neat PBS. Figure 12b shows a similar result. At a constant isothermal crystallization temperature, the overall crystallization rate goes through a maximum at 0.5 wt% MWCNTs and then progressively decreases. Note that with 4 wt% MWCNTs (and 23 wt% PC) the overall crystallization rate is five times lower than that of neat PBS.

#### 3.5.1. Fitting DSC Isothermal Data to the Avrami Model

**Figure 13:**
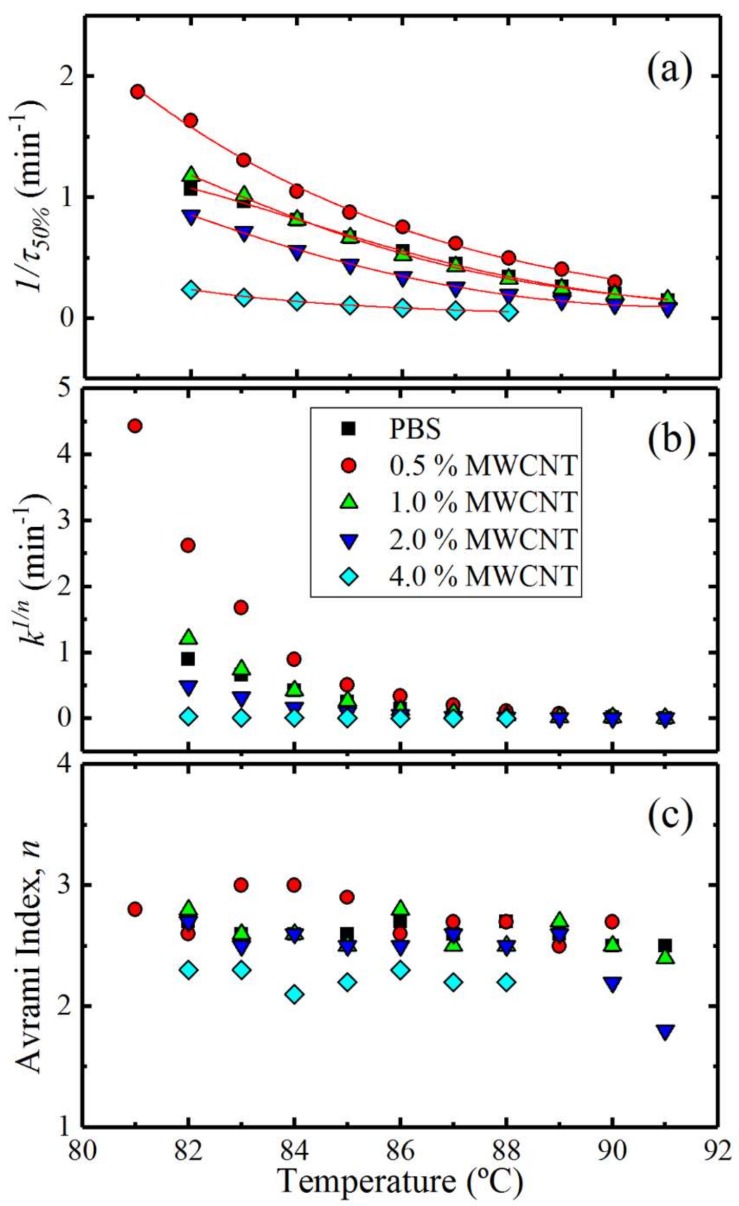
(**a**) Overall half-crystallization rate (the solid lines indicate the Lauritzen and Hoffman fitting); (**b**) Normalized crystallization constant of the Avrami model (*k*^1/*n*^); (**c**) Avrami index (*n*) as a function of the isothermal crystallization temperature (*T_c_*) for all the samples.

The data obtained during the isothermal crystallization experiments were analysed employing the Avrami equation, which can be expressed as Equation (4) according to ref. [25].

(4) $$1-V_{c}\left(t-t_{0}\right)=\exp(-k{\left(t-t_{0}\right)}^{n})$$

where t is the experimental time, $t_{0}$ is the induction time, $V_{c}$ is the relative volumetric transformed fraction, n is the Avrami index, and k is the overall crystallization rate constant. The procedure used to perform the fittings to the data was developed by Lorenzo et al. [25]. The kinetic parameters for all the investigated samples are plotted in Figure 13 and tabulated in Table S1 of the Supporting Information.

Figure 13a shows the experimental 1/*τ*_50%_ values as a function of *T_c_*, and the observed trend was explained earlier in the discussion (Section 3.5). The same trend is obtained when the Avrami rate constant (*k*) is normalized by elevating it to the power *n* (i.e., *k*^1/*n*^), and plotting it as a function of temperature (Figure 13b). This indicates that the fitting of the Avrami theory to the DSC data is consistent with the experimental results. In fact, the fitting was excellent in the primary crystallization range and examples of the comparison between experimental and fitted DSC isotherms can be found in the Supplementary Information (see Figure S3). Figure 13c shows the *n* values for all the samples, which depend on the dimensionality of the crystalline superstructure and on their nucleation kinetics [25,41].

The Avrami index values (*n*) obtained are within the range of 2.5–3.0 for all the samples in the investigated *T_c_* range, except for the 4 wt% MWCNTs containing nanocomposite. Avrami index values close to 3 indicate spherulitic morphology with instantaneous nucleation. For the 4 wt% MWCNTs nanocomposite, the value of *n* ranged between 2.0 and 2.3. Values lower than 3 are unexpected, especially when it has been demonstrated that MWCNTs are not effective in nucleating PBS at this content. It is well known that upon the addition of a nucleating agent, one would expect that the Avrami index would remain around 3 or would decrease (as the dimensionality of growth can switch from 3D to 2D when the nucleation density is greatly enhanced) [25,38,41]. In the present case, the nanocomposites with 4 wt% MWCNTs exhibit a higher degree of aggregation and the dispersion in the matrix is rather poor. The PBS-rich phase contains large aggregates of MWCNTs where the surrounding PBS chains may have a lower growth dimensionality (i.e., 2D or even 1D) as a result of being embedded within such large aggregates. The PBS chains away from the aggregates can still form spherulitic structures (3D) and since the overall kinetics takes into account all of the crystallizing PBS chains, it is possible that the resulting Avrami index falls below 2.5, as observed.

#### 3.5.2. Overall Isothermal Crystallization Data Analysed by the Lauritzen–Hoffman Model

The overall crystallization kinetics is determined by contributions of nucleation and growth. The Lauritzen–Hoffman (LH) nucleation and growth theory can be applied to the isothermal crystallization kinetics data collected from DSC. Even though the LH theory has received much criticism lately [42], it is still one of a few models that provides easy-to-use analytical expressions capable of fitting the experimental data over a wide supercooling range [43]. Figure 11 shows solid lines that represent the mathematical fit of the LH theory, which can be applied to the DSC overall crystallization data according to Equation (5).

(5) $$\frac{1}{\tau_{50\%}}\left(T\right)=\frac{1}{\tau_{0}}\exp\left(\frac{-U^{*}}{R(T_{c}-T_{\infty )}}\right)\exp\left(\frac{-K_{g}^{\tau }}{T_{c}\Delta T_{f}}\right),$$

where 1/*τ*_50%_ is the inverse of the experimental half-crystallization time, 1/*τ*_0_ is a pre-exponential factor that includes nucleation and growth, U* is the activation energy for the transport of the chains to the growth front (a value of 1500 cal·mol^−1^ is usually employed), *R* is the gas constant, *T_c_* is the isothermal crystallization temperature (K), *T*_∞_ is the temperature at which chain mobility ceases (usually taken as *T_g_* − 30 K), Δ*T* is the supercooling ($T_{m}^{o}-T_{c}$), $T_{m}^{o}$ is the equilibrium melting temperature calculated for each blend (see Table 2), and $K_{g}^{\tau }$ is a constant related to the energy barrier for crystallization and growth. The value of $K_{g}^{\tau }$ is given by Equation (6) according to the LH theory.

(6) $$K_{g}^{\tau }=\frac{jb_{0}\sigma \sigma_{e}T_{m}^{0}}{k\Delta h_{f}},$$

where *b*_0_ is the width of the chain, *σ* is the lateral surface free energy, *σ_e_* is the fold surface free energy, *k* is the Boltzmann constant and Δ*h_f_* is the heat of fusion of a perfect crystal. The parameter *j* is determined by the operating regime and was taken as 2 for regime II (note that *j* is equal to 4 for regime I and III). The product *σσ_e_* is obtained from the values of $K_{g}^{\tau }$, according to Equation (6). The following expressions allow the calculation of *σ* (and therefore *σ_e_*) and *q*, the work done by the chain to form a fold.

(7) $$\sigma =0.1\Delta h_{f}\sqrt{a_{0}b_{0}}$$

(8) $$q=2a_{0}b_{0}\sigma_{e}$$

where *a*_0_*b*_0_ is the cross-sectional area of the chain. To obtain the parameters of the LH theory, the following values were used: *T_g_* = 213 K, *T_g_* − 30 K, Δ*H* = 163 J g^−1^, *a*_0_ = 4.52 Å, *b*_0_ = 4.12 Å, *p_c_* = 1.1 g cm^−3^, U* = 1500 cal mol^−1^.

The LH parameters for neat PBS and the nanocomposites are tabulated in Table 3. There is a slight variation of $K_{g}^{\tau }$ values with increasing masterbatch content. This is in line with the already discussed *T_g_* values.

### 3.6. Thermal Conductivity

**Figure 14:**
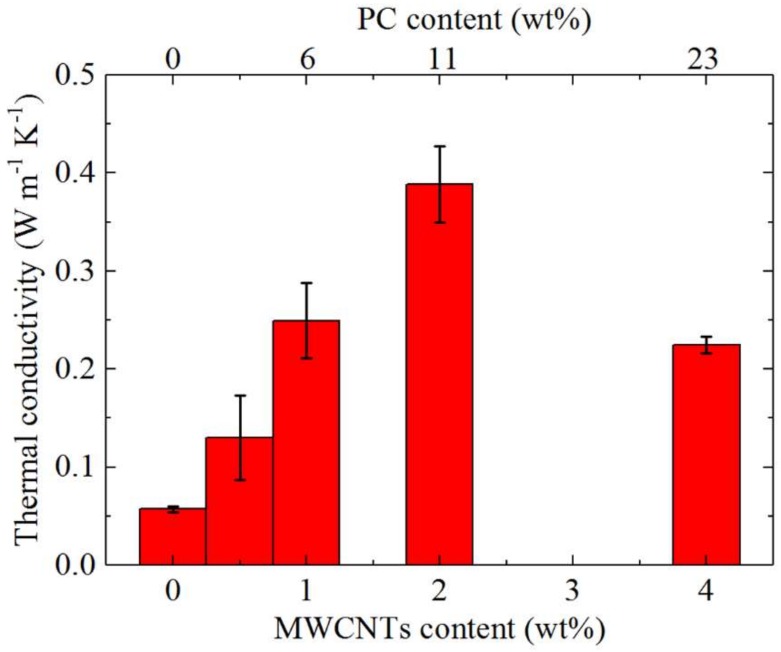
Influence of MWCNTs content on the thermal conductivities of the nanocomposites. The PC content is indicated in the top x-axis.

Figure 14 shows the thermal conductivities of neat PBS and its PC/MWCNTs containing nanocomposites. Phonon transport is the main mechanism for heat conduction in conductive polymer samples. Phonons transfer heat energy through interactions with each other and with subatomic particles. In multi-phase systems, such as polymeric composites, scattering also occurs when phonons propagate through a boundary which separates one phase from another [44,45,46]. Generally, there is an improvement in the thermal conductivity of the blend nanocomposites in comparison to neat PBS. Possible explanations for the improvement in thermal conductivity of the nanocomposites include: (1) the high thermal conductivity of the carbon nanotubes (650 to 10,000 W·m·K^−1^) [43], and (2) the dispersion of the filler particles in the polymer blend.

For the lowest amount of nanotubes (0.5 wt%), there are too few nanotubes for phonons to move effectively, hence the low thermal conductivity value. As the amount of nanotubes increased, there was an increase in the effectiveness of the movement of the phonons through the nanocomposites up to 2 wt% MWCNTs. Above this content, there was a decrease in the thermal conductivity. This is because above this content, the MWCNTs aggregated in the PC-rich phase and became ineffective parts of the PBS matrix. The phonons could move well in the PC-rich phase, but the fairly large PBS-rich phase areas that separated the PC-rich phase areas considerably slowed down the phonon movement, giving rise to reduced thermal conductivity.

### 3.7. Tensile Properties

**Figure 15:**
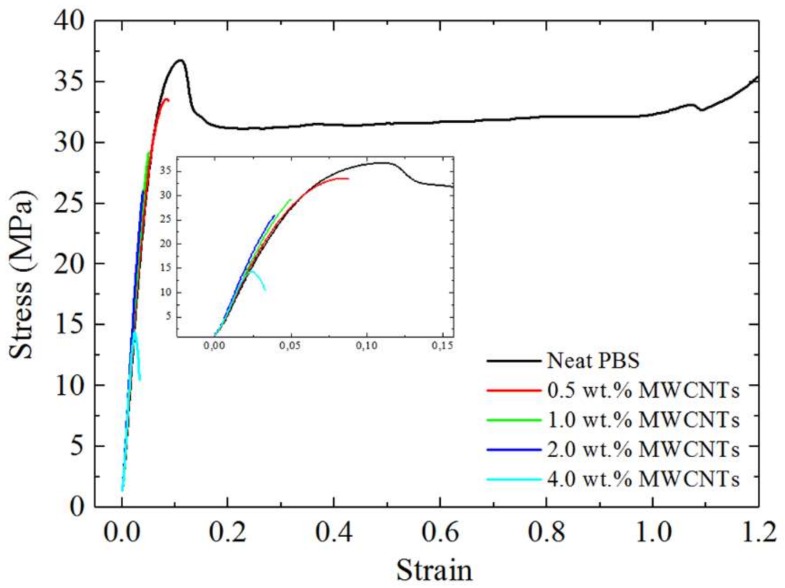
Stress-strain curves for neat PBS and the nanocomposites.

The mechanical properties of neat PBS and the nanocomposites were investigated through tensile testing. Figure 15 shows typical stress-strain curves obtained for the samples, whereas Table 4 summarizes the tensile test results. PBS is a ductile material which becomes fragile with the incorporation of the MWCNTs masterbatch.

The stress at break shows a decrease with the incorporation of the masterbatch (Figure 15). However, the nanocomposites containing 0.5, 1.0, 2.0 wt% MWCNTs gave almost the same value. The initial drop in tensile stress with the inclusion of the masterbatch in the PBS matrix is the result of the formation of the sea island morphology, with the very large dispersed PC-rich phase acting as a stress concentration region. PBS loses its localized shear deformation ability to form a neck and instead becomes a fragile material, as cracks nucleate at the dispersed phase and grow to produce earlier fracture. As a consequence, the strain at break dramatically drops when the masterbatch is added.

The tensile stress, however, remains fairly high for the samples containing up to 2 wt% MWCNTs, which could be related to the partial miscibility of the blends, where in spite of the large size of the dispersed PC-rich phase, there is adhesion between the PC-rich and the PBS-rich phases, as manifested in the lack of void formation at the interphase between the phases, as documented by SEM and AFM. For the 4 wt% MWCNTs nanocomposite, there was a very large drop in the tensile stress. This is probably due to the MWCNTs aggregates within the PC-rich phase (in addition to the PC-phase crystallization) that result in an increasing chance that the polymer will fracture at a much lower stress.

Table 4 shows little change in the Young’s modulus with increasing masterbatch content, taking into account the experimental error indicated by the standard deviation values. PBS had a modulus value of 579 ± 139 MPa, and this value remained virtually constant with increasing masterbatch content, despite the fact that the masterbatch had a Young’s modulus value of approximately 2800 MPa. There are two possible reasons why the modulus of the blend nanocomposites did not increase as expected: (1) The poor dispersion of the MWCNTs in the PBS-rich phase, and (2) the high level of nanoparticle aggregation in the PC-rich matrix [47].

## 4. Conclusions

The results reported in this paper showed that the PBS/masterbatch blends were partially miscible and formed PC-rich and PBS-rich phases, while the majority of the MWCNTs were inside the PC-rich phase. PBS plasticization caused some PC crystallization. The MWCNTs were able to nucleate the PBS-rich phase at low loading contents (below 4%), indicating that some of the MWCNTs were able to transfer from the PC-rich to the PBS-rich phase, as visualized by AFM. However, when the content of MWCNTs reached 4%, the PBS-rich phase was antinucleated. This was explained by the agglomeration of MWCNTs, which remained encapsulated inside the PC-rich phase, and the decreased ability of the PBS chains to nucleate. Such lower nucleation density may have arisen by a combination of reasons: impurities transfer from the PBS-rich to PC-rich phase and increased *T_g_* value of the PBS-rich phase in comparison to neat PBS. The isothermal crystallization rate also increased with low contents of MWCNTs, went through a maximum and then decreased in a consistent way with the non-isothermal results.

The thermal conductivities and tensile properties of the nanocomposites varied but could generally be explained in terms of the observed morphology of the nanocomposites. For example, a significant decrease in thermal conductivity was observed for the 4 wt% MWCNTs content, which probably was the result of a high level of nanoparticle aggregation in the PC-rich matrix. This was probably also the reason for the low stress-at-break value for this particular sample, which resulted in enhanced stress concentrations leading to early fracture.

The last part of this study will focus on PCL-PBS blends that we plan to mix with the PC-MWCNT masterbatch. This study will be conducted along the same lines as the work done in the present paper, but it will be interesting to see the influence of the presence of low-melting PCL on the morphology and crystallization behaviour of respectively PCL and PBS. This will obviously be determined by the extent to which the MWCNTs disperse into this tri-polymer system.

## Figures and Tables

**Table 1:** Weight percentages of the components in the nanocomposites.

PBS (wt%)	PC (wt%)	MWCNTs (wt%)
100	0	0
97	2.55	0.45
93	5.95	1.05
87	11.05	1.95
73	22.95	4.05

**Table 2:** Calculated values of *d*-spacing (from WAXS experiments) and long period (d*, obtained from SAXS experiments) for the neat PBS and its nanocomposites.

Sample	*q* (nm^−1^)/*d*-spacing (nm)/(Plane)	d* (nm)
Neat PBS	13.88/0.426/(002) 15.54/0.381/(012) 15.96/0.371/(110) 18.38/0.322/(121) 20.36/0.291/(111)	12.3
93/(6/1) *w*/*w* PBS/(PC/MWCNTs)	13.90/0.426/(002) 15.52/0.381/(012) 15.97/0.371/(110) 18.35/0.323/(12-1) 20.42/0.291/(111) 12.34/0.479 *	11.9 **
73/(23/4) *w*/*w* PBS/(PC/MWCNTs)	13.88/0.426/(002) 15.55/0.381/(012) 15.95/0.371/(110) 18.34/0.323/(121) 20.36/0.291/(111) 12.30/0.481 *	8.3 **

* PC signal; ** overlap of PC and PBS signals.

**Table 3:** Parameters from the isothermal crystallization kinetics analyses for neat PBS and the PBS/(PC/MWCNTs) nanocomposites.

Sample	$T_{m}^{o}$(K)	$K_{g}^{\tau }$ × 10^4^ (K^2^)	*σ* (erg/cm^2^)	*σ_e_* (erg/cm^2^)	*q* × 10^−13^ (erg)	*R*^2^
100/0	394.0	8.16	8.08	81.13	2.99	0.995
97/(2.5/0.5)	393.0	8.01	8.08	79.83	2.95	0.998
93/(6/1)	392.1	7.91	8.08	79.04	2.92	0.999
87/(11/2)	391.1	6.99	8.08	70.01	2.58	0.987
73/(23/4)	389.1	6.79	8.08	68.32	2.52	0.997

**Table 4:** Summary of tensile testing results for neat PBS and the nanocomposites.

*w*/*w* PBS/(PC/MWCNTs)	$\sigma_{b}$ (MPa)	$\varepsilon_{b}$ (%)	*E* (MPa)
100/0	38.1 ± 2.1	210 ± 110	579 ± 139
97/(2.5/0.5)	28.3 ± 6.1	7.0 ± 2.9	641 ± 50
93/(6/1)	29.4 ± 0.5	5.0 ± 0.1	672 ± 44
87/(11/2)	26.0 ± 6.6	5.0 ± 1.0	715 ± 84
73/(23/4)	10.9 ± 0.6	4.0 ± 0.8	638 ± 79

$\sigma_{b}$—stress at break; $\varepsilon_{b}$—strain at break; *E*—Young’s modulus.

## Supplementary Materials

The following are available online at http://www.mdpi.com/2073-4360/10/4/424/s1.
