# Supplementary material for: Morphology, Nucleation, and Isothermal Crystallization Kinetics of Poly(Butylene Succinate) Mixed with a Polycarbonate/MWCNT Masterbatch
Source: Polymers (Basel). 2018 Apr 10;10(4):424. doi: 10.3390/polym10040424 (PMC6415459; doi:10.3390/polym10040424)
Supplement: Supplementary file 1 [file polymers-10-00424-s001.pdf]

polymers

MDPI

Supplementary Materials

Morphology, Nucleation, and Isothermal Crystallization Kinetics of Poly(Butylene Succinate) Mixed with a Polycarbonate/MWCNT Masterbatch

Thandi P. Gumede [1], Adriaan S. Luyt [2,*], Ricardo A. Pérez-Camargo [3], Agnieszka Tercjak [4] and Alejandro J. Müller [3,5,*]

[1] Department of Chemistry, University of the Free State (Qwaqwa Campus), Private Bag X13, Phuthaditjhaba 9866, South Africa; tpgumede66@gmail.com
[2] Center for Advanced Materials, Qatar University, P.O. Box 2713, Doha, Qatar
[3] POLYMAT and Polymer Science and Technology Department, Faculty of Chemistry, University of the Basque Country UPV/EHU, Paseo Manuel de Lardizabal 3, 20018 Donostia-San Sebastián, Spain; riky0712@gmail.com
[4] Group "Materials + Technologies" (GMT), Department of Chemical and Environmental Engineering, Faculty of Engineering, Gipuzkoa, University of the Basque Country UPV/EHU, 20018 Donostia-San Sebastián, Spain; agatercjak@gmail.com
[5] IKERBASQUE, Basque Foundation for Science, 48013 Bilbao, Spain
* Correspondence: aluyt@qu.edu.qa (A.S.L.); alejandrojesus.muller@ehu.es (A.J.M.); Tel.: +974-4403-5677 (A.S.L.); +34-943018191 (A.J.M.)

Self-nucleation (SN) experiments

Figure S1 shows the experimental data obtained during an SN experiment for neat PBS. The cooling scans after the isothermal step at T_s are presented in Figure S1(a), and the subsequent heating scans are shown in Figure S1(b). The dashed line indicates the PBS crystallization and melting temperatures under standard conditions. The three SN domains are described below as defined by Fillon *et al.* [1,2].

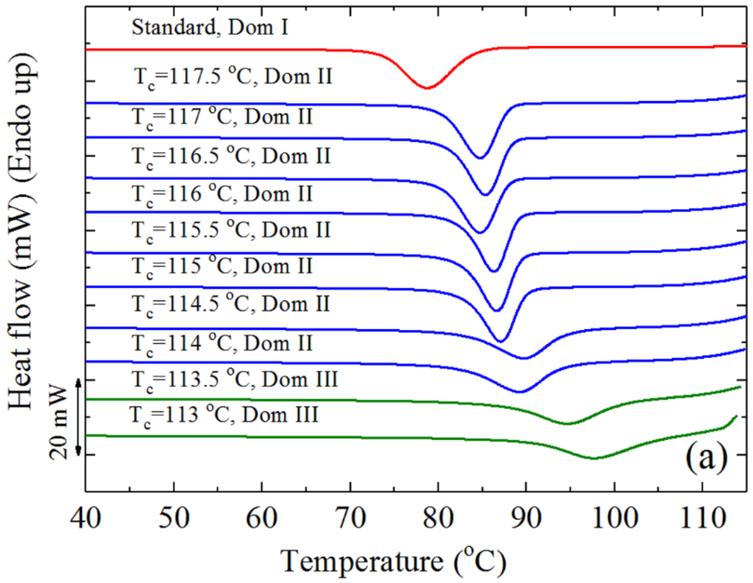

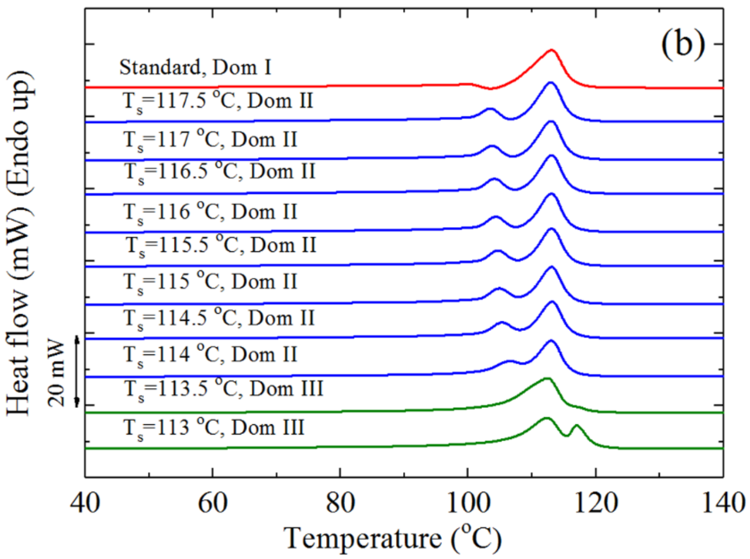

Figure S1. (**a**) DSC cooling scans for neat PBS after 5 min at the indicated T_s, and (**b**) subsequent heating scans after the cooling runs shown in (**a**).

Domain I or melting domain. The polymer is under *Domain I* when complete melting occurs and the crystalline history of the material is erased. For the PBS, *Domain I* is found at T_s equal to 120 °C, since no change was detected in the T_c when compared to the standard T_c. Both the crystallization and melting DSC scans are identical within *Domain I*.

Domain II or self-nucleation domain. In this domain, the T_s range employed is low enough to produce self-nuclei, but high enough to avoid annealing. Therefore, *Domain II* is easily identified after 5 min at a given T_s, because the peak crystallization temperature of the sample increases compared to the standard value. The start of *Domain II* for the PBS sample occurred at a T_s = 114 °C (Figure S1(a)), since the sample was self-nucleated without any annealing. The minimum T_s within *Domain II* is defined as the 'ideal self-nucleation temperature ($T_{s,ideal}$)', a temperature which should be accurately determined. This is the temperature that causes maximum self-nucleation (maximum increase in T_c) without annealing. The subsequent melting curve in Figure S1(b) does not reveal any sign of annealing. In this domain the nucleation density is greatly enhanced.

Domain III or self-nucleation and annealing domain. When T_s is too low, partial melting occurs and the unmolten crystals anneal during the 5 min at T_s. Figure S1(b) shows that at T_s < 114 °C the melting endotherm exhibits a small high temperature peak that is the result of the melting of the annealed crystals. At this T_s, the crystallization exotherm shows a high temperature tail which reveals that the sample is in *Domain III*.

Figure S2 shows the location of the three self-nucleation domains for the PBS sample. The vertical dashed lines indicate the temperatures at which the material experiences a self-nucleation domain transition [1,3]. Since 114 °C is the lowest T_s value in *Domain II*, it is called the ideal self-nucleation temperature, because it is the temperature at which there is maximum self-nucleation without any annealing. Employing the ideal T_s (114 °C), the T_c corresponding to the ideal T_s should be used as the maximum crystallization temperature ($T_{c,max}$) when determining the nucleation efficiency of the nanofiller. For the PBS used in this study, $T_{c,max}$ is 89.3 °C.

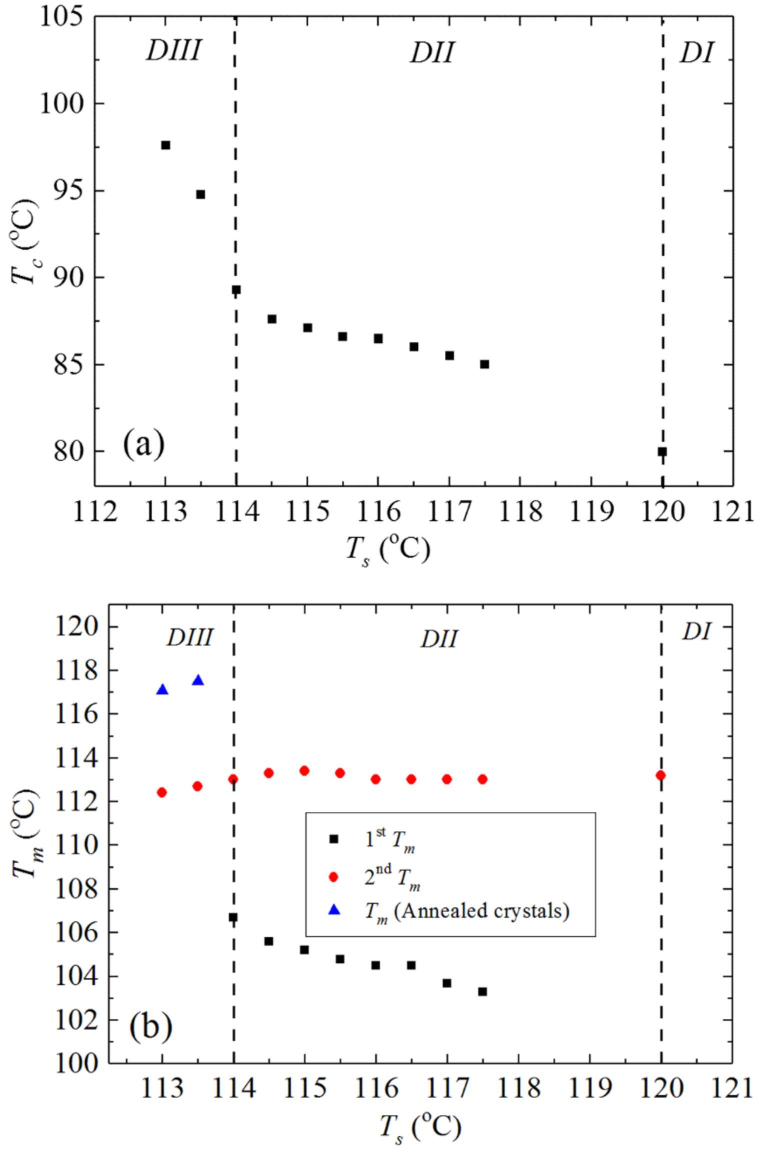

Figure S2. Dependence of (**a**) crystallization- and (**b**) melting peak temperatures of neat PBS on T_s.

Fitting of DSC isothermal data to the Avrami model

The data obtained by isothermal DSC tests were used to perform the Avrami fits and the graphical comparisons between the experimental data and the predictions of the theory.

An example of such a comparison is shown in Figure S3 for the 73/(23/4) w/w PBS/(PC/MWCNTs) isothermally crystallized at 83.0 °C, in which the experimental results and the corresponding Avrami prediction for the isothermally crystallized samples is shown. Figure S3(a) shows the data obtained from integration of the DSC isotherm and the vertical purple dashed lines indicate the integration range used. The vertical green dashed lines indicate the half crystallization time found experimentally. Figure S3(b) shows a plot of 1-V_c or the relative amorphous fraction as a function of crystallization time derived from an integration of the data in Figure S3(a). A typical sigmoidal shape describes the kinetics of transformation to the semicrystalline state. In this case the data is well described by the Avrami equation up to a conversion fraction of 0.8 (or 80%). Figure S3(c) shows the experimental data (circles) obtained from the isothermal crystallization and the solid line represents the Avrami fit. The normalised crystallization enthalpies as function of the crystallization time from the experimental results correlates well with the Avrami fit (Figure S3(d)). This indicates that the Avrami model predicts very well the isothermal crystallization.

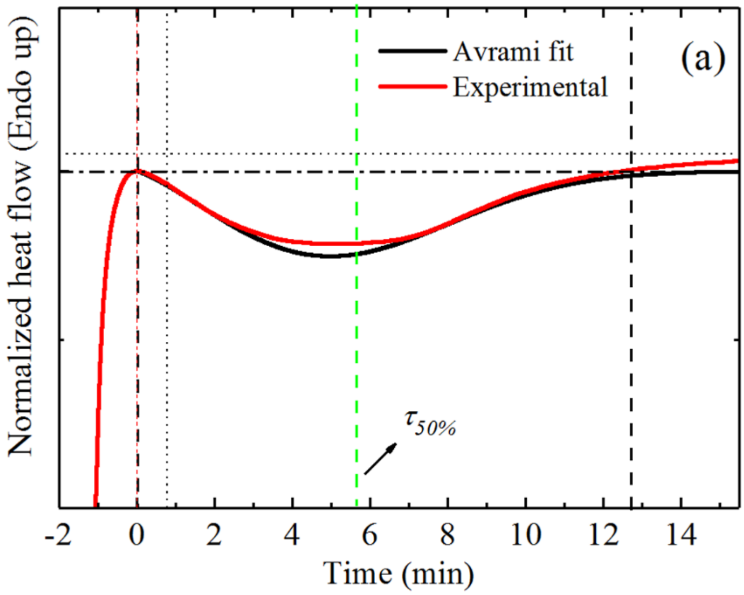

(a)
Avrami fit
Experimental
Normalized heat flow (Endo up)
Time (min)
τ50%
-2
0
2
4
6
8
10
12
14

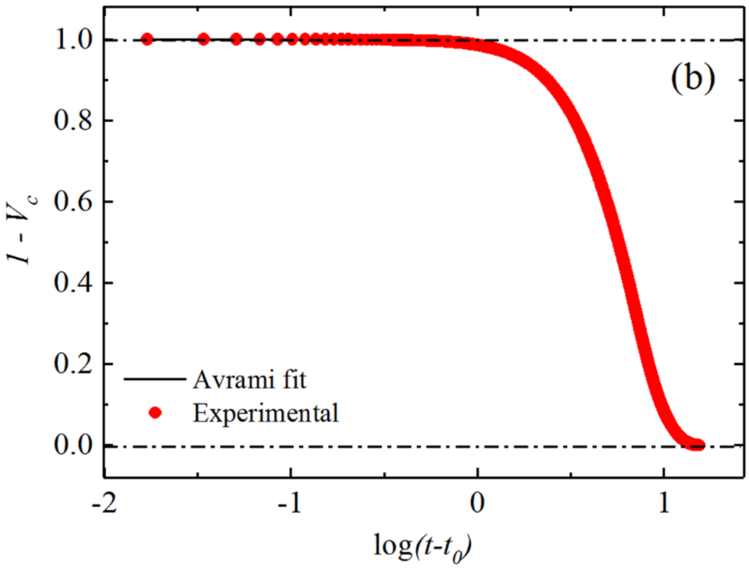

(b)
1 - Vc
1.0
0.8
0.6
0.4
0.2
0.0
Avrami fit
Experimental
-2
-1
0
1
log(t-t0)

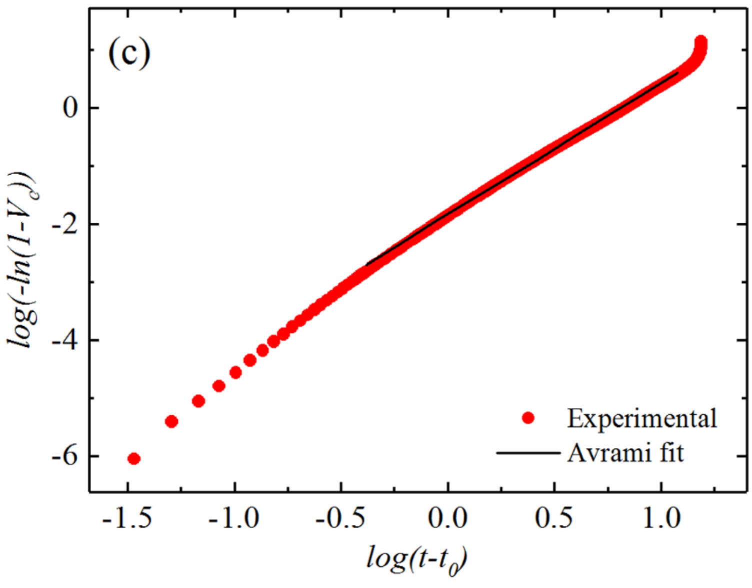

(c)
log(-ln(1-Vc))
0
-2
-4
-6
Experimental
Avrami fit
-1.5
-1.0
-0.5
0.0
0.5
1.0
log(t-t0)

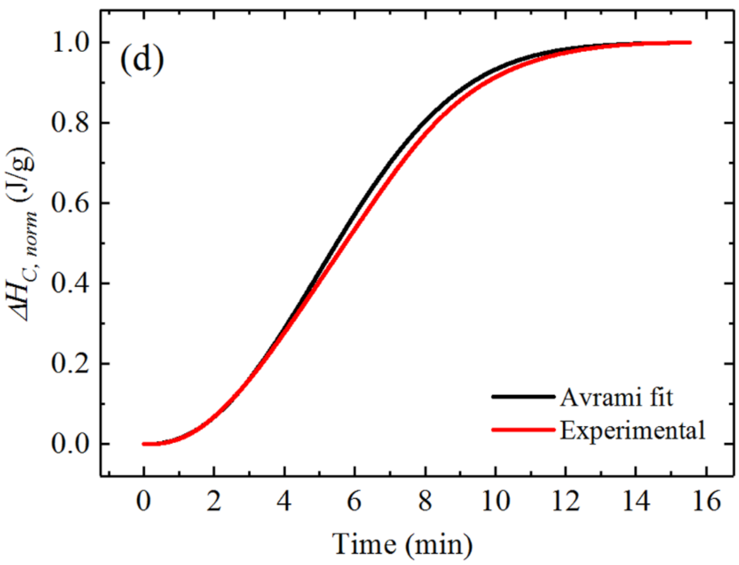

Figure S3. Comparison between experimental results and the corresponding Avrami prediction for a 73/(23/4) w/w PBS/(PC/MWCNTs) nanocomposite isothermally crystallized at 83.0 °C: (**a**) isothermal heat flow; (**b**) unconverted relative fraction; (**c**) Avrami plot; (d) normalized ΔH_c as a function of time.

The kinetic parameters for all the investigated samples are shown in Table S1. It is worth noting that a conversion range of approximately 3–20% was used and this corresponds to the primary crystallization range where the Avrami analysis is most adequate. In such a range the correlation coefficients of the fit are mostly in excess of 0.999 (Table S1). For all the samples studied, the half crystallization times for the experimental data ($\tau_{50\%\ Exp}$) and the Avrami fittings ($\tau_{50\%\ Theo}$) are almost the same, which indicates that the Avrami model predicts very well the crystallinity up to 50% relative crystallinity.

Table S1. Kinetic parameters for all the investigated samples during isothermal crystallization.

PBS/(PC/MWCNTs) Sample	T_c (°C)	t_o (min)	ΔH (J.g^{-1})	V_c range (%)	n	k (min^{-n})	R^2	$\tau_{50\%\ Theo}$ (min)	$\tau_{50\%\ Exp}$ (min)	$(\tau_{50\%\ Exp})^{-1}$ (min^{-1})
Neat PBS	82.0	1.46	30	3-20	2.7	9.05E-01	0.9999	0.91	0.94	1.0638
	83.0	1.58	33	3-20	2.6	6.62E-01	1.0000	1.02	1.04	0.9615
	84.0	1.62	37	3-20	2.6	4.27E-01	1.0000	1.20	1.22	0.8197
	85.0	1.68	40	3-20	2.6	2.57E-01	1.0000	1.46	1.50	0.6667
	86.0	1.72	41	3-20	2.7	1.49E-01	0.9999	1.76	1.80	0.5556
	87.0	1.84	49	3-20	2.6	9.19E-02	0.9999	2.16	2.21	0.4525
	88.0	1.89	53	3-20	2.7	4.17E-02	0.9998	2.81	2.92	0.3425
	89.0	1.99	57	3-20	2.6	2.23E-02	0.9999	3.76	3.84	0.2604
	90.0	2.13	61	3-20	2.5	1.24E-02	1.0000	4.97	5.00	0.2000
	91.0	2.18	64	3-20	2.5	6.19E-03	1.0000	6.58	6.63	0.1508
97/(2.5/0.5) w/w PBS/(PC/MWCNTs)	81.0	1.33	25	3-20	2.8	4.43E+00	1.0000	0.52	0.53	1.8868
	82.0	1.48	28	3-20	2.6	2.62E+00	1.0000	0.60	0.61	1.6393
	83.0	1.50	28	3-20	3.0	1.68E+00	0.9998	0.74	0.77	1.2987
	84.0	1.53	30	3-20	3.0	8.99E-01	0.9998	0.92	0.95	1.0526
	85.0	1.58	31	3-20	2.9	5.08E-01	0.9999	1.11	1.14	0.8772
	86.0	1.70	31	3-20	2.6	3.43E-01	1.0000	1.31	1.33	0.7519
	87.0	1.75	32	3-20	2.7	2.01E-01	1.0000	1.59	1.62	0.6173
	88.0	1.84	34	3-20	2.7	1.12E-01	0.9998	1.96	2.01	0.4975
	89.0	2.01	37	3-20	2.5	7.48E-02	1.0000	2.45	2.47	0.4049
	90.0	2.01	44	3-20	2.7	3.04E-02	0.9999	3.24	3.33	0.3003
93/(6/1) w/w PBS/(PC/MWCNTs)	82.0	1.51	19	3-20	2.8	1.21E+00	0.9999	0.82	0.85	1.1765
	83.0	1.70	19	3-20	2.6	7.40E-01	1.0000	0.98	0.99	1.0101
	84.0	1.79	21	3-20	2.6	4.23E-01	1.0000	1.21	1.22	0.8197
	85.0	1.92	22	3-20	2.5	2.63E-01	1.0000	1.47	1.50	0.6667
	86.0	1.94	22	3-20	2.8	1.24E-01	0.9996	1.84	1.92	0.5208
	87.0	2.11	24	3-20	2.5	8.65E-02	0.9999	2.28	2.33	0.4292
	88.0	2.21	27	3-20	2.5	4.35E-02	0.9999	2.97	3.04	0.3289
	89.0	2.28	17	3-20	2.7	1.81E-02	0.9997	3.86	4.01	0.2494
	90.0	2.60	30	3-20	2.5	1.25E-02	0.9999	4.93	5.05	0.1980
	91.0	2.98	31	3-20	2.4	7.87E-03	0.9999	6.27	6.40	0.1563

**87/(11/2) w/w PBS/(PC/MWCNTs)**	82.0	1.67	14	3-20	2.7	4.88E-01	0.9999	1.14	1.17	0.8547
	83.0	1.87	14	3-20	2.5	3.23E-01	0.9999	1.35	1.39	0.7194
	84.0	1.94	16	3-20	2.6	1.69E-01	0.9997	1.71	1.79	0.5587
	85.0	2.06	17	3-20	2.5	1.02E-01	0.9999	2.19	2.24	0.4464
	86.0	2.18	19	3-20	2.5	5.43E-02	0.9999	2.83	2.91	0.3436
	87.0	2.21	21	3-20	2.6	2.22E-02	0.9994	3.68	3.91	0.2558
	88.0	2.36	24	3-20	2.5	1.29E-02	0.9997	4.84	5.07	0.1972
	89.0	2.33	27	3-20	2.6	5.88E-03	0.9998	6.50	6.77	0.1477
	90.0	2.18	31	3-20	2.2	5.57E-03	0.9997	8.89	8.72	0.1147
	91.0	2.16	40	3-20	1.8	7.43E-03	0.9990	12.83	11.46	0.0873
**73/(23/4) w/w PBS/(PC/MWCNTs)**	82.0	2.30	30	3-20	2.3	2.99E-02	0.9998	4.00	4.18	0.2392
	83.0	2.43	36	3-20	2.3	1.47E-02	0.9998	5.48	5.73	0.1745
**73/(23/4) w/w PBS/(PC/MWCNTs)**	84.0	2.77	36	3-20	2.1	1.09E-02	1.0000	6.95	7.07	0.1414
	85.0	2.94	36	3-20	2.2	5.57E-03	1.0000	8.92	9.04	0.1106
	86.0	2.94	34	3-20	2.3	2.73E-03	1.0000	11.64	11.71	0.0854
	87.0	3.32	33	3-20	2.2	1.64E-03	1.0000	14.96	14.84	0.0674
	88.0	3.59	27	3-20	2.2	1.05E-03	0.9999	19.84	18.48	0.0541

References

1. Fillon, B.; Wittmann, J.C.; Lotz, B.; Thierry, A. Self-nucleation and recrystallization of isotactic polypropylene (α phase) investigated by differential scanning calorimetry. *J. Polym. Sci. B: Polym. Phys.* **1993**, *31*, 1383–1393, doi:10.1002/polb.1993.090311013.
2. Michell, R.M.; Mugica, A.; Zubitur, M.; Müller, A.J. Self-nucleation of crystalline phases within homopolymers, polymer blends, copolymers, and nanocomposites. *Adv. Polym. Sci.* **2015**, *276*, 215–256. doi:10.1007/12_2015_327.
3. Lorenzo, A.T.; Arnal, M.A.; Sánchez, J.J.; Müller, A.J. Effect of annealing time on the self-nucleation behaviour of semicrystalline polymers. *J. Polym. Sci. B: Polym. Phys.* **2006**, *44*, 1738–1750, doi:10.1002/polb.20832.

© 2018 by the authors. Submitted for possible open access publication under the terms and conditions of the Creative Commons Attribution (CC BY) license (http://creativecommons.org/licenses/by/4.0/).
